# All Biomass and UV Protective Composite Composed of Compatibilized Lignin and Poly (Lactic-acid)

**DOI:** 10.1038/srep43596

**Published:** 2017-03-08

**Authors:** Youngjun Kim, Jonghwan Suhr, Hee-Won Seo, Hanna Sun, Sanghoon Kim, In-Kyung Park, Soo-Hyun Kim, Youngkwan Lee, Kwang-Jin Kim, Jae-Do Nam

**Affiliations:** 1 School of Chemical Engineering, Department of Polymer Science and Engineering, Sungkyunkwan University, Suwon 440-746, South Korea; 2 Department of Energy Science, Sungkyunkwan University, Suwon 440-746, South Korea; 3 Center for Biomaterials, Korea Institute of Science and Technology, Seoul 136-791, South Korea; 4 Department of Chemical Engineering, Sungkyunkwan University, Suwon 440-746, South Korea; 5 Department of Mechanical Engineering, University of Nevada Las Vegas, 4505 S, Maryland Parkway, Box 454027, Las Vegas, NV, 89154-4027, USA

## Abstract

Utilization of carbon-neutral biomass became increasingly important due to a desperate need for carbon reduction in the issue of global warming in light of replacing petroleum-based materials. We used lignin, which was an abundant, low cost, and non-food based biomass, for the development of all biomass-based films and composites through reactive compatibilization with poly (lactic-acid) (PLA). Using a facile and practical route, the hydrophilic hydroxyl groups of lignin were acetylated to impose the compatibility with PLA. The solubility parameter of the pristine lignin at 26.3 (J/cm^3^)^0.5^ was altered to 20.9 (J/cm^3^)^0.5^ by acetylation allowing the good compatibility with PLA at 20.2 (J/cm^3^)^0.5^. The improved compatibility of lignin and PLA provided substantially decreased lignin domain size in composites (12.7 μm), which subsequently gave transparent and UV-protection films (visual transmittance at 76% and UV protection factor over 40). The tensile strength and elongation of the developed composite films were increased by 22% and 76%, respectively, and the biobased carbon content was confirmed as 96 ± 3%. The developed PLA/lignin composites provided 100% all-biomass contents and balanced optical and mechanical properties that could broaden its eco-friendly applications in various industries.

Global warming caused by human-made carbon emissions is an important issue of today. Recently, the Paris Agreement within the United Nations Framework Convention on Climate Change (UNFCCC) starts in 2020 more seriously dealing with greenhouse-gas emissions aiming for holding the increment of the global average temperature to well below 2 °C^1^. The implementation of the signed agreement by all countries will be evaluated every 5 years on the basis of Intended Nationally Determined Contributions (INDCs). Since the carbon stemming from biomass is counted as an intrinsic zero carbon footprints, the demand for altering the carbon sources from petrochemical to biomass feedstock has been increased^2^. Recently, an accurate evaluation method of biomass-carbon content in the final products has been codified as an ASTM standard, D6866 titled “Standard Test Methods for Determining the Biobased Content of Solid, Liquid, and Gaseous Samples Using Radiocarbon Analysis”^3^. The radiocarbon, (^14^C, half-life of 5,730 years) is created by collision with cosmic ray neutrons and ^14^N in the atmosphere and subsequently present in all the biomass materials through the photosynthesis during their lifetimes at a concentration of one-trillion of ^12^C^4^,^5^. It now allows determining the biomass carbon content of materials or parts within ±3% in accuracy measuring the amount of ^14^C^3^.

Using this method, the biobased carbon content of various bioplastics can be accurately evaluated and gives somewhat surprising results. For example, cellulose acetate, which is commercially available and well known bioplastic, gives around 50% of biobased carbon content^6^. Commercial biodegradable plastic, poly (ε-caprolactone) (PCL) is a completely petro-based plastic giving 0% of biobased carbon content^7^. Another commercialized bioplastic, bio-polyethylene terephthalate (Bio-PET), which is synthesized from bio-ethylene glycol (31.25 wt%) and petro-based terephthalic acid (68.75 wt%), gives only 20% of biobased content^8^. This is because of the fact that the carbon content of petro-based terephthalic acid is higher than bio-based ethylene glycol. Thus, it should be mentioned that the increment of biomass content and the subsequent decrement of CO_2_ emission in many plastic products is still a challenging issue in the time era of facing global warming of the earth.

Poly (lactic-acid) (PLA) is 100% biomass polyester polymerized by lactic-acid mainly derived from corn starch and it is currently one of the most extensively-used bioplastics with a volume of 180,000 ton/year in 2015^9^. It is commonly used in biodegradable packaging industry^10^. As for packaging material, UV protection capability is often an essential property for the protection of inside goods. The UV protection materials include titanium dioxide, zinc oxide and organic UV absorbers such as phenylbenzotriazole and dibenzoylmethances^11^. Incorporation of those UV-protection materials into the PLA matrix usually decreases transparency^12^ and biobased content in the final products. Natural products such as green coffee oil, extracts of carica papaya, rosa kordesii, helichrysum arenarium, etc. also have UV protection functions^13^. However, those natural products have limited capability in UV protection that cannot block the full spectrum of UV light. In addition, the extraction of active ingredients from raw materials is expensive and their large volume commercial production is limited yet.

Lignin, the most abundant biomass containing aromatic rings in nature, accounts for 20–30% of wood by weights^14^,^15^. Lignin has hyperbranched structure with various functional groups, such as hydroxyl, methoxyl, ether, and aldehyde groups^15^. It is also known as a natural broad-spectrum UV blocker due to such functional groups as phenolics, ketone and other chromophores^16^,^17^,^18^. It should be noted that lignin protects the UV-vulnerable cellulose fibers during the life time of plants in nature. Most industrial lignin is produced as a waste during the paper pulping process in a large volume of approximately 50 million tons worldwide, positively ensuring its mass supply as a biomass resource at a low cost^19^. The aromatic structure of lignin gives high carbon content at 61–66 wt%^20^. Lignin is a 100% natural biomass, abundant, low cost, and non-food based resource and, thus, it can be an ideal material for the development of biomass-based UV protective composite materials. Recently, the UV absorbance property of lignin has been reported^13^. However, when lignin is incorporated over 10 wt%, the sunscreen effect disappears due to the ease of lignin agglomeration resulting in a short shelf-life time and low-level sun blocker. Therefore, lignin dispersion and compatibilization with matrix materials are considered to be one of the key issues for the development of novel lignin-based UV-protection composite materials.

Most of all the natural lignin (~98%) is not currently being used as value-added products but discarded as an industrial waste due to its natural form of vulnerable chemical structures, i.e., lack of resistance against heat, chemicals, external loading, etc. Natural lignin tends to aggregate in polymer composites due to the π-π stacking of its aromatic rings, hydrogen bonding among hydroxyl groups, and poor compatibility with other polymers, which usually impairs the properties of the resulting lignin composites^21^,^22^. To overcome these drawbacks, several chemical modification routes have been investigated such as acetylation, propionation, butyration, and maleation using hydroxyl groups in lignin^23^. Particularly, acetylation reaction has been adopted in the modification of many biopolymers including cellulose, starch, and natural fiber^24^,^25^,^26^. Acetylation replaces hydrophilic hydroxyl groups with hydrophobic acetyl groups consequently converting hydrophilic lignin to hydrophobic one. It desirably decreases the hydrogen-bond strength in lignin molecules and subsequently gives a decreased domain size of aggregated lignin when it is mixed with organic polymers.

Lignin has been incorporated into PLA to develop biomass based composites. For example, hydrolytic degradation^14^, antioxidant activity^27^, and mechanical properties^28^,^29^ of PLA/lignin composites has been investigated exhibiting large-sized immiscible domains and impairment of mechanical properties due to poor compatibility of lignin with PLA. Therefore, we herein developed a novel methodology that ensures the enhanced compatibility and the domain-size control of PLA/lignin composites, which have been the key issues in the successful development of eco-friendly UV-protective lignin/PLA composite materials. In the present work, the 100% all-biomass composite was developed using the kraft lignin feedstock recovered from the waste of pulping industry. The solubility parameter of lignin was adjusted by the acetylation reaction to become comparable with PLA. The lignin domain size in the PLA matrix was investigated by morphology analysis. The optical, mechanical, thermal, and barrier properties of the developed lignin/PLA composites were investigated clearly demonstrating their reliable and wide applicability capable of reducing carbon emission and global warming as well as recycling harmful wastes.

## Results and Discussion

### Determination of lignin acetylation

**Figure 1:**
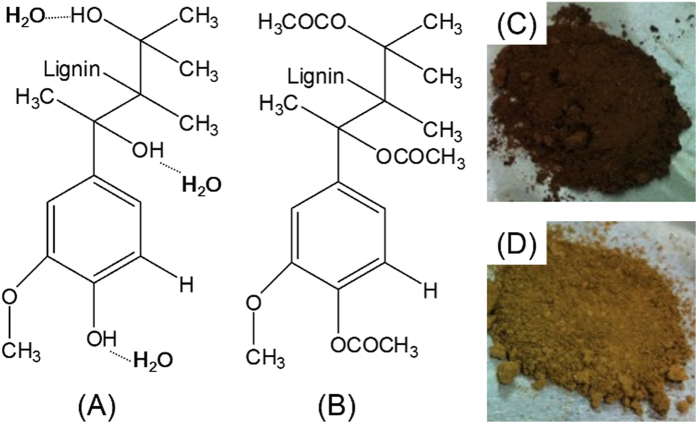
Monolignol chemical structures of (**A**) LIG and (**B**) a-LIG. Camera images of (**C**) dried LIG and (**D**) a-LIG powder.

Figure 1A and B schematically show the monolignol structure of the pristine lignin (LIG) and the acetylated lignin (a-LIG), respectively. LIG is a dark brown wet powder that seems to easily absorb moisture in the air seemingly due to the abundant hydroxyl groups in lignin (Fig. 1C). However, a-LIG is a bright brown dry powder due to the fact that the hydrophilic hydroxyl groups are reduced by the acetylation reaction (Fig. 1D). A strong intermolecular hydrogen bonding of LIG leads to self-aggregation of powders and water uptake, which is not the case with a-LIG.

**Figure 2:**
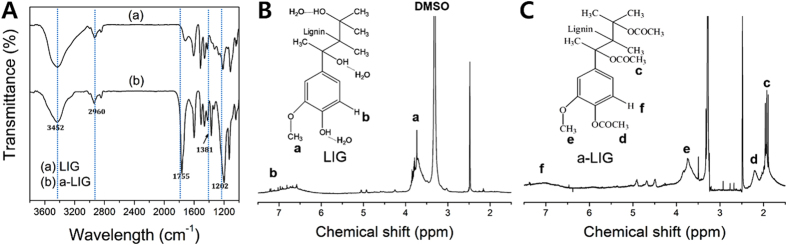
Characterization of LIG and a-LIG. (**A**) Functional groups of the LIG and a-LIG analyzed by FT-IR, (**B** and **C**) ^1^H-NMR results indicating chemical structure of the LIG and a-LIG using DMSO-d_6_.

The characterization and analysis of lignin are usually a complex procedure because its structure, molecular weight, functionality, etc. depend on both its natural origin and the way it was separated from the plants^30^. Through acetylation, the hydroxyl groups of lignin are substituted with acetyl groups, which makes the lignin hydrophobic and soluble in some organic solvents. Figure 2 compares the FT-IR and ^1^H-NMR spectra of LIG, a-LIG. Their FT-IR spectra absorption bands and ^1^H-NMR chemical shifts are summarized in Tables 1 and 2, respectively. In Fig. 2A, the peak of aromatic alkyl chain of lignin is centered at 2960 cm^−1^ (methoxyl group), which is intact for both LIG and a-LIG^31^. In both spectra of LIG and a-LIG, two strong bands also exist at around 3452 cm^−1^ (OH stretching), where LIG exhibits higher intensity than a-LIG due to the acetylation reaction. The characterization spectra of a-LIG are observed by the presence of three important non conjugated carbonyl bonds at 1755 cm^−1^ (C=O ester), 1381 cm^−1^ (C-H bond in an –O(C=O)-CH_3_ group), and 1202 cm^−1^ ((-CO-) stretching of acetyl group)^32^. The absence of absorption bands in the region of 1840–1760 cm^−1^ in a-LIG indicates that all the acetic anhydride is consumed by the acetylation reaction. The lack of a carboxylic group peak at 1700 cm^−1^ in a-LIG also implies that the products are free of the byproduct of acetic acid.

Figure 2(B and C) show the ^1^H-NMR spectra of LIG and a-LIG, respectively. Using NMR, it is possible to estimate the amount of both aliphatic and phenolic hydroxyl groups^33^. As reported in previous studies, a-LIG leads to better signal resolution, lower signal overlapping, and proton coupling effects^34^. The signal at 2.5 ppm is related to DMSO-d_6_. Methoxy protons (-OCH_3_) give an intense signal centered at 3.8 ppm. All the signals ranging from 6.0 to 7.5 ppm are attributed to aromatic protons in guaiacyl units of LIG molecules. In a-LIG, aliphatic and aromatic groups of the acetyl protons are clearly shown at 1.9 and 2.2 ppm, respectively, which confirms the acetylation reaction. By integration of the signal area, the hydroxyl group content can be estimated, where the total hydroxyl group content is 10.09 mmol/g and the aliphatic/aromatic hydroxyl group ratio is 3.67.

### Solubility parameter analysis of LIG and a-LIG

The interactions among molecules may be evaluated using the solubility parameters, which are calculated by dispersion forces, polar forces, and hydrogen bonding^35^. When two molecules have approximately equal solubility parameters, they tend to be soluble with each other. The solubility parameters for polymers are calculated by accounting for the contributions of the cohesive energy and molar volume of each functional group and fragment of the main structure of the molecules^36^. According to the group contribution method (GCM), the solubility parameter (δ) is calculated as follows:


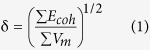


where δ is the solubility parameter of a molecule, E_coh_ is the cohesive energy of a functional group on the molecule, and V_m_ is its molar volume. The values of E_coh_ and V_m_ for each functional group of LIG and a-LIG are summarized in Table 3. The resulting solubility parameters of LIG and a-LIG are 26.31 (J/cm^3^)^0.5^ and 20.87 (J/cm^3^)^0.5^, respectively, as summarized in Table 4. The solubility parameters of chloroform (CF), which are used as a solvent for preparing specimens in this study, and PLA are 18.6 (J/cm^3^)^0.5^ and 20.2 (J/cm^3^)^0.5^, respectively. As can be realized, the solubility parameter of a-LIG (20.87 (J/cm^3^)^0.5^) is closer to that of CF and PLA than LIG (26.31 (J/cm^3^)^0.5^). It clearly indicates that the compatibility of a-LIG with CF and PLA is substantially improved.

### Compatibility analysis of LIG and a-LIG in organic solvents

**Figure 3:**
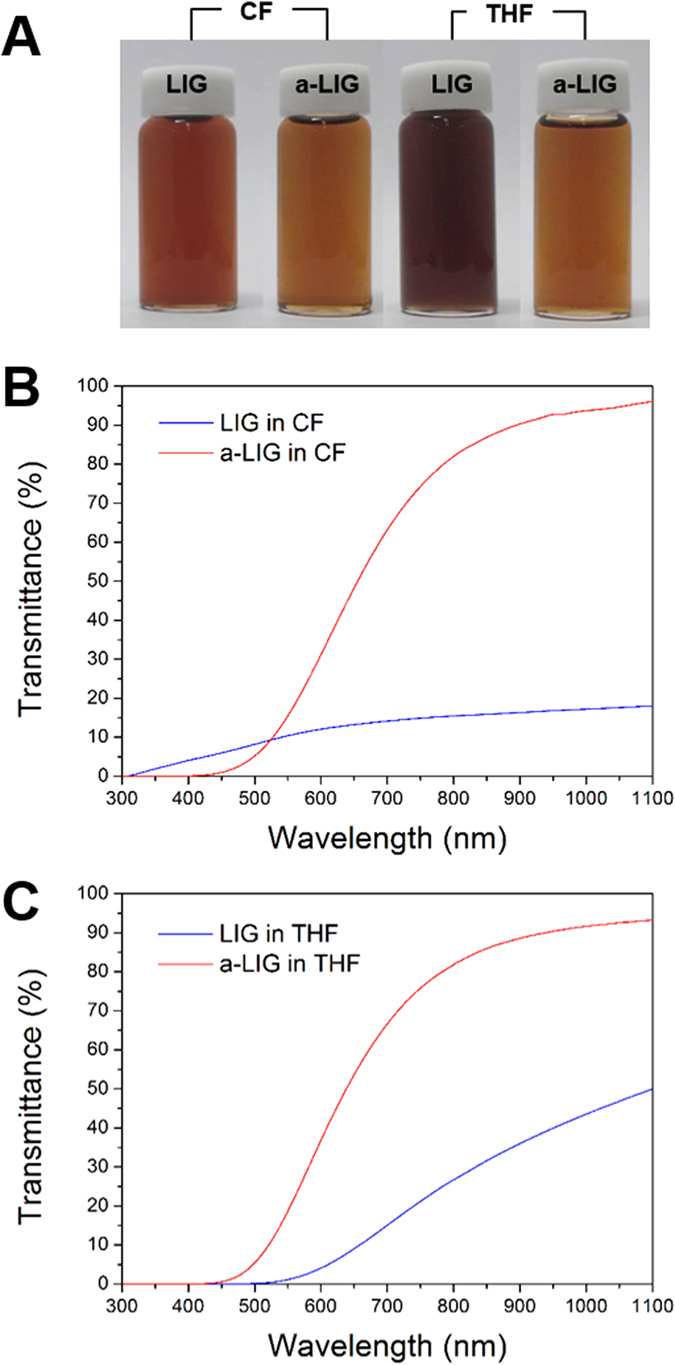
(**A**) Digital images of LIG and a-LIG dispersion (1 wt%) in CF and THF. UV-vis transmittance spectrums of LIG and a-LIG dispersion in (**B**) CF and (**C**) THF.

Figure 3 shows the visual observation and UV-vis transmittance spectra of LIG and a-LIG dispersed in THF and CF (1 wt%), respectively. In Fig. 3A, a-LIG dispersion is more transparent than the LIG dispersion. Figure 3(B and C) show that the transmittance of a-LIG dispersion is higher than that of the LIG dispersion in the visual light range (>400 nm), demonstrating enhanced solubility of a-LIG in both THF and CF. It is supported by the GCM analysis that the solubility parameter of a-LIG (20.87 (J/cm^3^)^0.5^) is closer to THF (18.2 (J/cm^3^)^0.5^) and CF (18.6 (J/cm^3^)^0.5^) than LIG (26.31 (J/cm^3^)^0.5^). In addition, it should be noticed that the UV transmittance (<400 nm) is effectively cut-off by the lignin, which seems to be due to the hyperchromic effect of the spectroscopic properties of the aromatic molecules of lignin in the UV regions^16^,^17^,^18^,^37^. This demonstrates the UV absorption capability of lignin.

### Biobased carbon content of PLA/lignin composite films

Biobased content of a material is the amount of biobased carbon in the material as fraction weight of the total organic carbon in the material. The ASTM D6866 set allows to calculate the biobased content using a ratio of the amount of radiocarbon (^14^C) in an unknown sample to that of a modern reference standard, i.e., oxalic acid^6^ without use of the age equations. Using the ^14^C concentrations, ^14^As (^14^C/^12^C of specimen) and ^14^Ar (^14^C/^12^C of reference), measured by Accelerator Mass Spectroscopy (AMS), the biobased contents can calculated as follows:


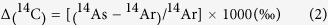



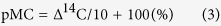



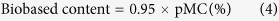


**Figure 4:**
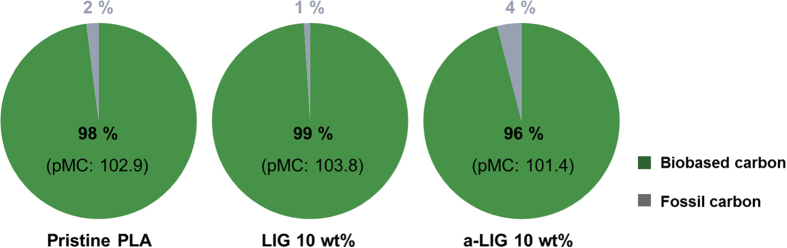
Biobased carbon content of pristine PLA, PLA/LIG, and PLA/a-LIG composite films measured by ASTM D6866.

Δ(^14^C) is the isotope differential ratio of ^14^C in the sample and reference material, and pMC is the percent of modern carbon. In this standard method, the pMC of biomass is adjusted as 95% (atmospheric correction factor) due to the diminishing effects nuclear testing programs in the 1950s. Using these equations, the biobased carbon contents of pristine our specimens of PLA, PLA/LIG, and PLA/a-LIG containing LIG and a-LIG at 10 wt% are estimated as shown in Fig. 4. The pMC values for pristine PLA, PLA/LIG, and PLA/a-LIG are 102.9, 103.8, and 101.4, respectively, and the resulting biobased content values are 98%, 99%, and 96%, respectively. Since both PLA and lignin are made of renewable biomass, it clearly demonstrates that PLA/lignin composite films can be regarded as 100% biobased composites.

### The aggregation size and distribution of lignin in the PLA composite films

**Figure 5:**
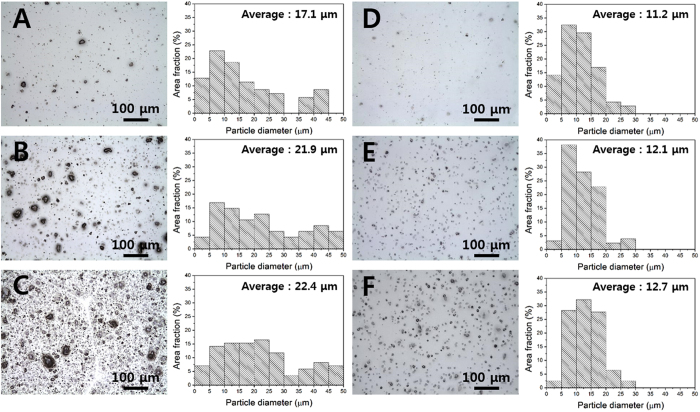
Optical images and size distribution histogram of LIG and a-LIG aggregates in PLA/LIG and PLA/a-LIG composite films at different LIG and a-LIG concentration: (**A** and **D**) 1 wt%, (**B** and **E**) 5 wt%, and (**C** and **F**) 10 wt%.

The optical morphology and the size distribution histogram of the PLA/LIG and PLA/a-LIG are shown in Fig. 5. As can be seen, PLA appears as a white and transparent matrix domain and lignin as a dark brown island dispersed in the PLA matrix. The size of primary particles of lignin has been determined to be about 3.5 nm^38^. In fact, the size of lignin aggregates in PLA matrix depend on the compatibility between lignin and PLA as with most other polymers, where the size of lignin aggregates decreases with enhanced compatibility. The average diameters of lignin aggregates of PLA/LIG containing 1, 5, and 10 wt% of LIG, are 17.1 μm, 21.9 μm, and 22.4 μm, respectively, which should be compared with 11.2 μm, 12.1 μm, and 12.7 μm, respectively, for PLA/a-LIG. Overall, it is clear that the average aggregate size of a-LIG is smaller than that of LIG when mixed with PLA as a composite form. It is also shown that the average size of pristine lignin, LIG aggregates, increases with LIG contents, while that of acetylated lignin, a-LIG aggregates, is nearly constant at 11.9 ± 0.7 μm. It clearly demonstrates that a-LIG has a good compatibility with PLA in composites. The size distribution histograms of lignin aggregates can be calculated using the area fraction of the aggregates in the optical images, and it is shown in the histograms (Fig. 5). The pristine lignin (LIG) shows a relatively wide spread and bimodal distribution of aggregates containing an exceptionally-large size over 30 um (Fig. 5A through C), while the acetylated lignin (a-LIG) shows uniform-sized and mono-dispersed distribution. It is clear that the large-sized lignin aggregates disappear by the acetylation reaction in PLA/a-LIG composites, which stems from the decreased hydrogen-bond strength and increased compatibility in lignin molecules. Since the acetylation treatment of lignin provides good compatibility with PLA and gives small size aggregation of lignin, it may ensure enhanced optical, mechanical, and processing-related characteristics in the PLA/lignin composite systems.

### Mechanical and thermal properties of PLA/LIG and PLA/a-LIG composite films

**Figure 6:**
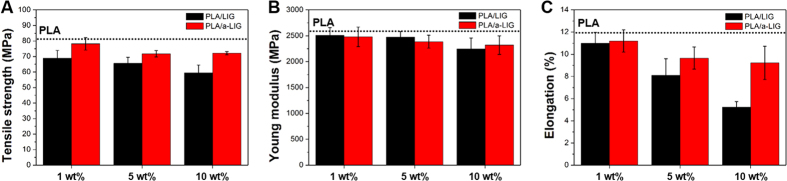
Tensile properties of PLA/LIG and PLA/a-LIG composite films. (**A**) The tensile strength, (**B**) Young’s modulus and (**C**) elongation at break of PLA/LIG and PLA/a-LIG composites with various LIG and a-LIG contents.

The mechanical properties (tensile strength, Young’s modulus, and elongation at break) of PLA/LIG and PLA/a-LIG composite films are compared in Fig. 6. The mechanical properties slightly decrease with the incorporation of lignin. Comparing LIG and a-LIG composite films, the mechanical properties of a-LIG composites are always higher than LIG ones in all the range of incorporated lignin content. The impairment of mechanical properties of PLA/LIG stems from large aggregates of lignin, which is minimized by acetylation reaction in PLA/a-LIG due to the improved compatibility of lignin with PLA. It is clearly confirmed that acetylation reaction endows PLA/lignin composite materials with moderate mechanical properties, which could possibly allow larger amount of a-LIG incorporation than the pristine lignin.

PLA is has a slow crystallization rate and poor heat resistance, and thus it is considered important to increase the crystallization rate of PLA. The glass transition temperature (T_g_), cold crystallization temperature (T_cc_), and melting temperature (T_m_) of the composite films are summarized in Table 5 (DSC thermograms in Supplement Figure S1). As can be seen, T_g_ is intact at 59 °C with the incorporation of either LIG or a-LIG meaning that there is not chemical changes of PLA main backbone chains stemming from chemical reactions. The T_m_ of the PLA film decreases with the incorporation of LIG and a-LIG, which is a typical T_m_ behavior observed in miscible blend systems of crystalline polymers^39^. The T_cc_ values of the composite films decreases by the addition of LIG or a-LIG indicating that the crystallization of PLA is facilitated by the presence of lignin aggregates. This may be due to the heterogeneous nucleation induced by the introduction of lignin^40^. The T_cc_ values of PLA/a-LIG are lower than that of PLA/LIG seemingly due to more active nucleating capability of a-LIG than LIG. As can be realized, it is also due to the fact that the enhanced compatibility of a-LIG gives smaller aggregates and larger interface area in a-LIG than the pristine lignin.

### Barrier properties of PLA/LIG and PLA/a-LIG composite films

Since PLA is an important candidate material for the applications in biodegradable and eco-friendly packaging films, the gas barrier properties are considered important. In this study, the water vapor transmission rate (WVTR), oxygen transmission rate (OTR), and water contact angle results of pristine PLA, PLA/LIG and PLA/a-LIG are measured and summarized in Table 6. The WVTR of PLA/LIG is higher than PLA since the pristine lignin is hydrophilic. However, the WVTR decreases from 5.09 g/m^2^·day of pristine PLA to 4.86 g/m^2^·day of PLA/a-LIG, which may very well give enhanced packaging characteristics. OTR and contact angles of lignin-incorporated PLA specimens are almost intact compared to the pristine PLA as 12.9+/−0.1 g/m^2^·day and 73.4+/−0.5°, respectively.

### Optical properties and UV-protection of PLA/LIG and PLA/a-LIG composite films

**Figure 7:**
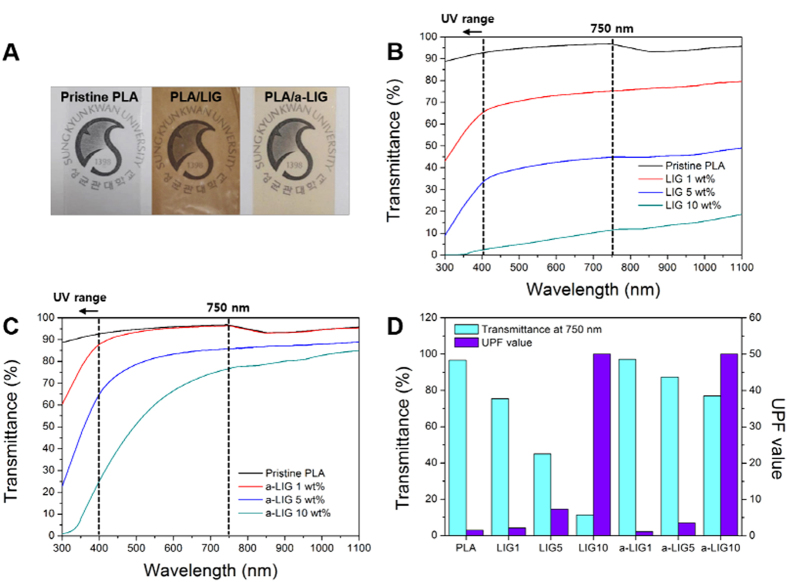
Optical properties of PLA/LIG and PLA/a-LIG composite films. (**A**) Camera images and UV-vis transmittance curves of (**B**) PLA/LIG and (**C**) PLA/a-LIG with various contents of LIG and a-LIG. (**D**) Visible transmittance at 750 nm and UPF value of the composite films with thickness of 30 μm.

Figure 7 shows the camera images (magnification of ×200) and UV-vis transmittance spectra of PLA/lignin composite films. As seen in Fig. 7A, the PLA/LIG film is opaque and dark brown while the PLA/a-LIG film is nearly transparent and bright yellow. In Fig. 7B and C compare the pristine and acetylated lignin, the PLA/a-LIG composite films give higher transmittance than PLA/LIG composite films in all the visual light range. Although, lignin is originally dark brown powder because lignin usually exists in the aggregated forms. When the size of the lignin aggregates become less than the wavelength of visible-light wavelength, however, the PLA/lignin film can be almost transparent^41^. Accordingly, it is clear that decreased size of a-LIG aggregates enhances transmittance of the film in visible light. As with the previous observation in organic solvents (Fig. 3), the transmittances of both PLA/LIG and PLA/a-LIG composite films decrease rapidly in the UV range (<400 nm). It proves that lignin can absorb the UV light. The UV protection capacity of PLA/LIG and PLA/a-LIG composite films is compared in Fig. 7D. According to the UV protection factor (UPF, AS/NZS 4399)^42^, the samples can be rated as providing good 15 < UPF < 24 (93.3 to 95.8% UV blocked), very good in 24 < UPF < 39 (95.9 to 97.4% UV blocked), and excellent protection in UPF > 39 (higher than 97.5% UV blocked). The UPF factors of both LIG and a-LIG composite with 10 wt% incorporation are over 40, which means excellent UV protection with higher than 97.5% UV blocked. Comparing the transmittance value at 750 nm (10 wt% of lignin), PLA/a-LIG shows 76% of transparency, which should be compared with PLA/LIG at 11% demonstrating that the PLA/a-LIG gives both excellent transparency and UV barrier property due to the enhanced compatibility with PLA. It demonstrates the potential applicability of PLA/a-LIG as an attractive all-biomass packaging and coating materials containing excellent transparency and UV-protection capability particularly important for light-sensitive products or human skins.

## Conclusion

All biomass-based composite films using lignin and PLA are developed by solvent casting technique. The biobased carbon content of PLA/lignin composite films clearly demonstrate that it can be regarded as 100% biobased composites. The solubility parameter of the lignin has been successfully controlled by acetylation reaction to enhance the compatibility with PLA, which gives the small aggregates size and uniform dispersion of a-LIG. Consequently, the mechanical properties and heterogeneous nucleation of PLA/lignin composite films are enhanced by acetylation due to the enhanced compatibility and increased interface area between lignin and PLA. The incorporation of hydrophobic a-LIG slightly improves the WVTR of PLA. The visual transmittance of PLA/lignin composite films is considerably increased by acetylation reaction. More interestingly, the PLA composite film at 10 wt% of a-LIG shows excellent UV protection factor (over 40) with high transmittance which demonstrate the potential application for biodegradable packaging materials.

## Methods

### Materials

Kraft lignin (M_n_ ~ 600, M_w_ ~ 1200) was achieved from a black liquor, which is composed of 35~45% of lignin, 40~45% of salts, and 10~15% of other organics. The black liquor that we used in this study was provided by Moorim Pulp & Paper Co. (South Korea) and we extracted kraft lignin from black liquor using our own method^43^. Acetic anhydride, pyridine, tetrahydrofuran (THF), and chloroform (CF) were purchased from Sigma-Aldrich. Poly (lactic-acid) (PLA) was a 2003D grade purchased from Natureworks Co., MN, USA.

### Synthesis of acetylated lignin

The oven-dried lignin (1 g) was mixed with acetic anhydride-pyridine (3:10, v/v, and 13 mL) and vigorously stirred for 24 hours at room temperature. The mixture was added dropwise in cold water and precipitated followed by centrifugation. The resulting solid product was thoroughly washed with DI water to remove the pyridine, unreacted acetic anhydride, and acetic acid byproducts. The samples were then dried overnight in an oven at 40 °C.

### Preparation of PLA/lignin films

The PLA/lignin films were prepared using solvent casting. PLA (7 g) was dissolved in 100 mL of chloroform with vigorous stirring at room temperature (RT). Lignin was added after complete dissolution of PLA. The solutions were stirred for another 1 h prior to casting. The solutions were then cast onto a 15 cm diameter glass petri dish, and then allowed to dry for a day at RT. The solvent cast films were placed in a vacuum oven at 40 °C for 1 week in order to remove all remaining chloroform. The prepared composite films had a thickness of 30+/−5 μm.

### Standard analysis methods for determining biobased content of carbon of PLA/lignin composite films

A biobased content of PLA/lignin composite films was analyzed by accelerator mass spectrometry (AMS) with ASTM D6866. The testing was performed at the BETA Analytic Inc., located in Miami, Florida using a 4130-Tandetron AMS. The carbon in graphite, transferred from samples, was ionized using a cesium cation beam. The anionized carbons were accelerated using AMS. The amounts of ^12^C and ^13^C were detected as a current using multi-Faraday cups. The ^14^C atoms were detected using a solid state detector with a semiconductor absorber. The concentration of ^14^C to ^12^C (^14^As) for PLA/lignin composite sample was calculated from the measured amounts of ^14^C and ^12^C.

### Characterization

The infrared spectra of lignin, acetylated lignin, and the PLA/lignin composite films were obtained using Fourier transform infrared (FT-IR) spectroscopy (Bruker IFS-66/S). ^1^H-NMR spectra of lignin and acetylated lignin were obtained by nuclear magnetic resonance (NMR) spectroscopy (Varian UNITY INOVA 500). Optical properties of films were measured using an ultraviolet-visible/near-infrared (UV-VIS/NIR) spectrometer (Varian Cary 5000). The morphology of the films was analyzed using an optical microscope (Nikon Eclipse Ni-E). Tensile tests were carried out by ASTM D638 using a universal testing machine (UTM, 4210 model, Instron Engineering). The crystallization behavior of the films was analyzed using differential scanning calorimetry (SEICO INST DSC 7020). The oxygen and water vapor transmission rate (OTR, WVTR) values of the films were determined using Mocon Oxtran 2/21 and Mocon Permatran-W 3/33, respectively. Contact angle measurements of the films were carried out using GBX Digidrop Contact Angle Meter.

## Additional Information

**How to cite this article:** Kim, Y. *et al*. All Biomass and UV Protective Composite Composed of Compatibilized Lignin and Poly (Lactic-acid). *Sci. Rep.* **7**, 43596; doi: 10.1038/srep43596 (2017).

**Publisher's note:** Springer Nature remains neutral with regard to jurisdictional claims in published maps and institutional affiliations.

## Supplementary Material

Supplementary Information

## Figures and Tables

**Table 1:** FT-IR spectra absorption bands of LIG and a-LIG.

Frequency (cm^−1^)	Possible assignment
3452	OH stretching
2960	Methoxyl group
1755	C=O stretching (ester bond)
1381	C-H bending (ester bond)
1202	C-O stretching (ester bond)

**Table 2:** ^1^H-NMR chemical shifts of selected protons.

Structure or functional group	Chemical shift (ppm)
Aromatic H	7.5-6.0
-OCH_3_	4.2-3.6
DMSO-d_6_	2.5
Aromatic acetate	2.6-2.2
Aliphatic acetate	2.2-1.6
TMS	0.0

**Table 3:** Cohesive energies and molar volumes used for the calculation of solubility parameters of LIG and a-LIG using group contribution method (GCM)^35^.

	Group	E_coh_ (J/mol)	V_m_ (cm^3^/mol)
LIG	Phenyl (trisubstituted)	31,940	33.4
	-CH3 × 5	23,550	167.5
	>C< × 3	4,410	−57.6
	-O- × 1	3,350	3.8
	-OH × 3	65,550	39.0
	Total	128,800	186.1
a-LIG	Phenyl (trisubstituted)	31,940	33.4
	-CH3 × 8	37,680	268.0
	>C< × 3	4,410	−57.6
	-O- × 1	3,350	3.8
	-COO- × 3	54,000	54.0
	Total	131,380	301.6

**Table 4:** Solubility parameters of LIG, a-LIG, CF, THF, and PLA used in this paper.

Solvents and polymers	δ (J/cm^3^)^0.5^
Lignin (LIG)	26.3
Acetylated lignin (a-LIG)	20.9
Poly (lactic-acid) (PLA)	20.2
Chloroform (CF)	18.6
Tetrahydrofuran (THF)	18.2

**Table 5:** Thermal properties of pristine PLA, PLA/LIG, and PLA/a-LIG composite films.

	T_g_ (°C)	T_cc_ (°C)	T_m_ (°C)
Pristine PLA	59.7	130.0	154.7
LIG 1 wt%	59.4	128.7	151.5
LIG 5 wt%	59.1	129.2	151.6
LIG 10 wt%	59.0	129.8	150.6
a-LIG 1 wt%	59.0	124.3	150.8
a-LIG 5 wt%	59.7	125.3	150.4
a-LIG 10 wt%	59.1	127.5	150.6

**Table 6:** Water vapor transmission rate (WVTR), oxygen transmission rate (OTR), and water contact angles of Pristine PLA, PLA/LIG, and PLA/a-LIG composite films.

	WVTR (g/m^2^·day)	OTR (g/m^2^·day)	Contact angle (°)
Pristine PLA	5.09	12.8	73.9
LIG 10 wt%	5.25	13.0	72.9
a-LIG 10 wt%	4.86	12.9	73.6
