# Supplementary material for: All Biomass and UV Protective Composite Composed of Compatibilized Lignin and Poly (Lactic-acid)
Source: Sci Rep. 2017 Mar 8;7:43596. doi: 10.1038/srep43596 (PMC5361212; doi:10.1038/srep43596)
Supplement: Supplementary Information [file srep43596-s1.pdf]

Supplemental information

All Biomass and UV Protective Composite Composed of Compatibilized Lignin and Poly (Lactic-acid)

Youngjun Kim[1], Jonghwan Suhr[1,2], Hee-Won Seo[1], Hanna Sun[2], Sanghoon Kim[2], In-Kyung Park[1], Soo-Hyun Kim[3], Youngkwan Lee[4], Kwang-Jin Kim[5], and Jae-Do Nam[1,2,*]

[1]School of Chemical Engineering, Department of Polymer Science and Engineering, Sungkyunkwan University, Suwon 440-746, South Korea

[2]Department of Energy Science, Sungkyunkwan University, Suwon 440-746, South Korea

[3]Center for Biomaterials, Korea Institute of Science and Technology, Seoul 136-791, South Korea

[4]Department of Chemical Engineering, Sungkyunkwan University, Suwon 440-746, South Korea

[5]Department of Mechanical Engineering, University of Nevada Las Vegas, 4505 S. Maryland Parkway, Box 454027, Las Vegas, NV 89154-4027, USA

[*]Correspondence and requests for materials should be addressed to J.D.N. (email: jdnam@skku.edu)

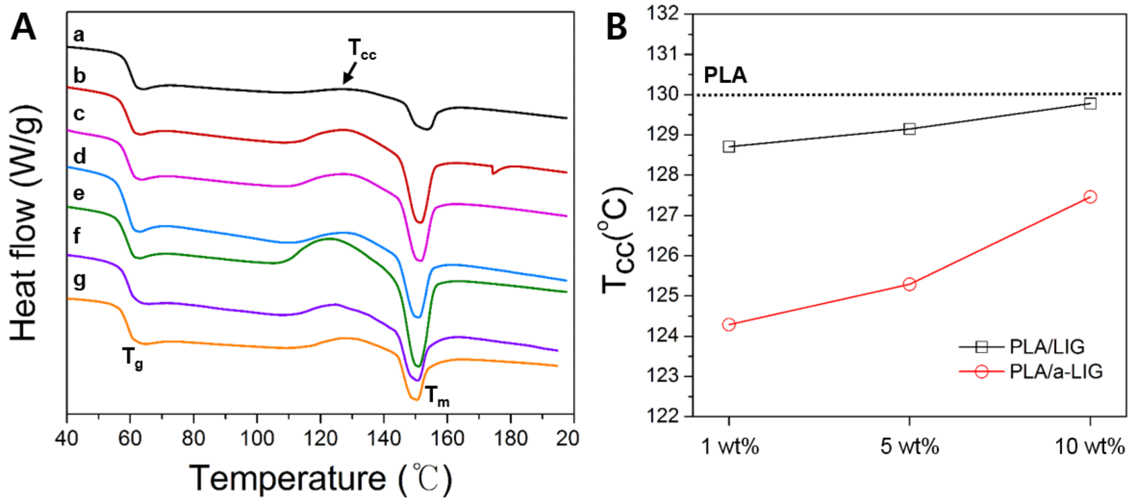

Supplemental Figure S1. DSC thermograms and T_{cc} values of PLA/LIG and PLA/a-LIG composite films: (A) The DSC curves of (a) pristine PLA, PLA/LIG and PLA/a-LIG containing LIG and a-LIG at (b, e) 1, (c, f) 5, and (d, g) 10 wt% by second heating scan (10℃/min). (B) The cold crystallization temperatures of the composite films.
